# Can Cervical Lymph Node Metastasis Increase the Risk of Distant Metastasis in Papillary Thyroid Carcinoma?

**DOI:** 10.3389/fendo.2022.917794

**Published:** 2022-06-24

**Authors:** Wenlong Wang, Ying Ding, Wei Jiang, Xinying Li

**Affiliations:** ^1^ Department of General Surgery, Xiangya Hospital, Central South University, Changsha, China; ^2^ National Clinical Research Center for Geriatric Disorders, Xiangya Hospital, Central South University, Changsha, China

**Keywords:** lymph node metastasis, papillary thyroid carcinoma, distant metastasis, N stage, age stratification

## Abstract

**Background:**

Distant metastasis (DM) is a rare event and has a negative effect on the prognosis for papillary thyroid carcinoma (PTC). The relationship between cervical lymph node metastasis and DM is complicated and unclear. This study aimed to evaluate the impact of N stage subclassification on different distant metastasis sites based on age stratification, especially for patients with papillary thyroid microcarcinoma.

**Methods:**

A total of 28,712 patient with PTC cases between 2010 and 2018 were extracted from the Surveillance, Epidemiology, and End Results database. Multivariable logistic regression analysis was utilized to adjust for confounding variables. Risk stratification, including positive lymph node number and lymph node ratio, was established by receiver operating characteristic curves to help predict DM.

**Results:**

Lung was the most common metastatic site regardless of N0, N1a disease, or N1b disease. As the N stage increased, the higher the rate of DM identified. After age stratification, only N1b disease significantly increased the risk of lung metastasis (LM; odds ratio, OR = 20.45, *P* < 0.001) rather than bone metastasis (BM; OR = 3.46, *P* > 0.05) in younger patients. However, in older patients, N1b disease significantly increased the risk of both LM (OR = 4.10, *P* < 0.001) and BM (OR = 2.65, *P* = 0.007). In patients with papillary thyroid microcarcinoma (PTMC), N1a disease did not increase the risk of DM, LM, and BM compared with N0 disease (*P* > 0.05). Furthermore, combined N stage with risk stratification has well performance in predicting DM (area under the curve, AUC = 0.761). Similar results were shown in PTC patients with LM (AUC = 0.770) and BM (AUC = 0.729).

**Conclusion:**

Overall, the incidence of DM significantly increased with the progress of N disease after age stratification. N1a disease did not increase the risk of DM in PTMC patients, regardless of LM or BM. Combined N stage with risk stratification may be beneficial for DM prediction.

## Introduction

Papillary thyroid carcinoma (PTC) was the most frequent histological subtype of thyroid malignancy which has a rapidly rising incidence worldwide in the last few decades (1, 2). In total, 20.7–62% of patients with PTC present cervical lymph node metastasis (LNM) at initial diagnosis, which was associated with a poor prognosis (3, 4), yet the recently updated 8th edition of the TNM staging system eliminates the difference between central (N1a) and lateral (N1b) LNM, and N1b disease is not adopted for the final staging classification (5–7). These changes have been questioned, and concern arises in such a way that ignoring the metastatic lymph node sites may underestimate recurrence and mortality in patients with PTC.

Distant metastasis (DM), including lung metastasis (LM), bone metastasis (BM), brain metastasis, and liver metastasis, contributes to the leading cause of thyroid cancer-related death (8), and the rates of 5- and 10-year cancer-specific survival (CSS) for PTC patients with DM were reported to be 35 and 25%, respectively (9, 10). Previous studies pointed out that LNM was considered as the strongest predictor of DM (11–13), especially in younger patients (<55 years) (14). Vuong and his colleagues (15) demonstrated that N1b disease was associated with a significant risk for DM. In contrast, N1a disease could not increase the risk of DM in patients with PTC. Surprisingly, further investigations on this relationship conducted by Wang (8) proved that LNM had no significant influence on DM. The conflicting results were partly attributable to neglecting the influence of potential confounding factors (advanced age, male gender, tumor size, and BRAF-V600E) on DM and simply exploring the risk factors that affect the overall DM without considering the heterogeneity of different metastatic sites. Thus, the role of LNM impact on DM has not reach an agreement (16), and there is no reference study focused on the effect of N stages subclassification on different metastatic sites.

The current study was undertaken to evaluate the effect of N classification on DM based on age stratification in patients with PTC using a large thyroid cancer cohort, especially for LM and BM. In the meantime, in order to guide the active surveillance (AS) for patients with papillary thyroid microcarcinoma (PTMC), we explore whether N1a disease can significantly increase the risk of DM. Furthermore, a DM predictive model based on N stages, positive lymph node number (PLNN), and lymph node ratio (LNR) was established to identify asymptomatic DM early. Therefore, the present results may provide guidance to personalize the therapeutic approaches and follow-up strategies.

## Materials and Methods

### Study Population

**Figure 1:**
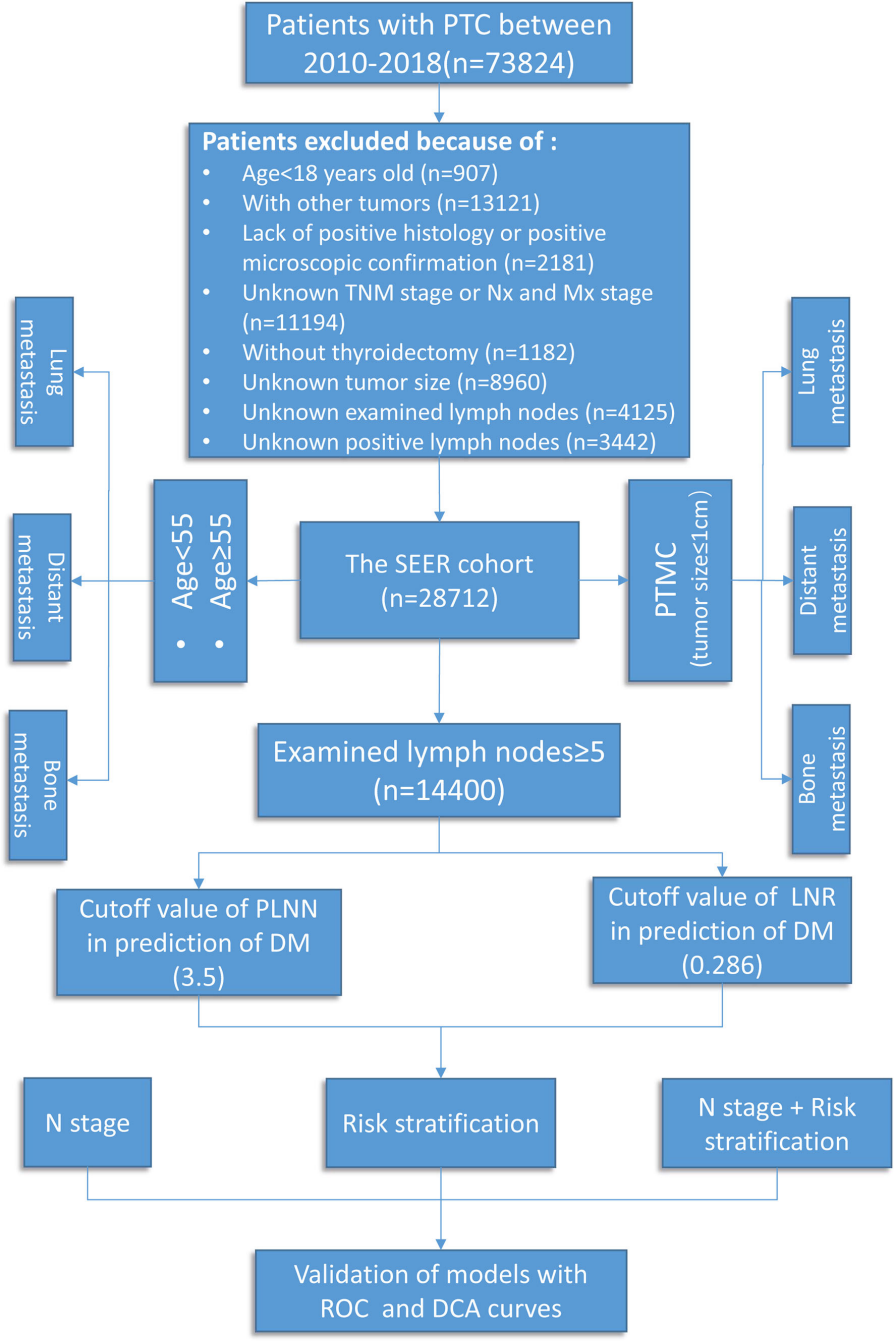
Flow chart of data screening and analysis.

All data were obtained from the Surveillance, Epidemiology, and End Results (SEER) database (https://seer.cancer.gov, access date: April 2021), a database that covered approximately 30% of the United States citizens and contained detailed demographics, therapy information, survival data, and clinicopathological characteristics (17). The International Classification of Diseases for Oncology code was used to identify patients diagnosed with PTC (8460, 8453, 8450, 8453, 8050, 8340-8344, and 8260) from the SEER 21 registry during the period 2010–2018. Data on age at diagnosis, T/N/M stage, grade (I, well differentiated; II, moderately differentiated; III, poorly differentiated; IV, undifferentiated, anaplastic), primary tumor size, distant metastatic sites, number of examined lymph nodes, PLNN, and radiation therapy (radioactive iodine therapy and external beam radiation therapy) were retrieved for further analysis. The inclusion criteria were as follows: (I) histologically confirmed PTC, (II) age ≥18 years, (III) clearly denoted N stage and distant metastatic sites (diagnosed by ultrasound, SPECT/CT, MRI, and post-treatment I131 whole-body scan during the 8 years of follow-up), and (IV) performed thyroidectomy with neck lymph node dissection. Patients with unknown or insufficient clinicopathologic profile were excluded. In total, 28,712 patients with PTC were enrolled in this study (**Figure 1**).

### Study Design

According to the 8th edition TNM-N classification system, the 28,712 PTC patients were classified into three groups: N0 (no evidence of LNM), N1a (metastasis to level VI or VII), and N1b (metastasis to retropharyngeal lymph nodes or level I – V) (18). The impact of N classification on DM was evaluated, especially for LM and BM. Recently, the revised 8th TNM stage updated the age cutoff from 45 to 55 years old; the effect of LNM on DM was analyzed by using age stratification, with a cutoff of 55 years. All patients were classified into the younger group (<55 years) and the older group (≥55 years) for further analysis. Finally, to evaluate the value of PLNN and LNR on DM, patients with retrieved lymph nodes less than 5 during neck lymph node dissection were ruled out to reduce the selection bias. LNR was defined as metastatic lymph nodes to total retrieved lymph nodes.

### Statistical Analysis

Statistical analyses were performed using R software (version 4.1.0). Continuous variables were presented as means ± standard deviations, while categorical variables were displayed as percentages and numbers. Univariate analysis was performed using *t*-test for continuous variables and chi-square test for categorical variables. To screen the independent risk factors affecting DM, continuous variables—including age and tumor size—and categorical variables—including T stage, race, sex, radiation therapy, grade, and laterality—were assessed by logistic regression analysis. In addition, odds ratios (OR) with 95% confidence intervals (CI) were calculated precisely. The area under the receiver operating characteristic (ROC) curve (AUC) was calculated to determine the most appropriate cutoff points for LNR and PLNN. Furthermore, a predictive model was constructed to categorize the patients into low-, medium-, and high-risk groups. Decision curve analysis (DCA) and AUC were utilized to assess the clinical performance and predictive accuracy. All *P*-values reported are two-sided, and those less than 0.05 were considered statistically significant.

## Results

### Baseline Clinical and Pathologic Characteristics

A total of 28,712 PTC patients were enrolled in this study, and the patient characteristics are presented in **Table 1**. The distribution of N classification was 13,973 (48.7%) with N0 stage, 9,251 (32.2%) with N1a stage, and 5,488 (19.1%) with N1b stage. Among these patients, 21,926 (76.4%) were female, and 72.4% were younger than 55 years old; the median age was 45.6 ± 14.5 years. Unilateral tumors, pathological tumor size ≤1 cm, and T1 stage were found in 20,442 (71.2%), 9,482 (33.0%), and 15,661 (54.5%) of the patients, respectively. About 38 (0.13%) patients underwent multiple distant metastasis. Besides this, patients with grade I/II (6,204) were significantly more numerous than patients with grade III/IV (242). The mean number of retrieved lymph nodes and PLNN were 10.7 ± 15.0 and 3.0 ± 5.4, respectively. About 15,173 (52.8%) patients underwent I131 therapy. The median follow-up duration was 61 months (range, 1–107 months), the 5-year overall survival and CSS were 98.3 and 99.2%, respectively. Besides this, DM was found in 319 (1.1%) patients; of them, 189 patients (0.65%) had LM, 63 (0.22%) had BM, 10 (0.03%) had liver metastases, and 9 (0.03%) had brain metastases. **Supplementary Figure S1** shows that lung is the most common metastatic sites regardless of N0, N1a disease, or N1b disease. As the stage increased, the higher the rate of DM was identified. Age, gender, grade, race, tumor size, PLNN, LNR, examined lymph nodes, T stage, M stage, radiation therapy, and survival time were significant difference among N classification (*P <*0.05).

**Table 1:** Baseline clinical and pathologic characteristics of patients.

Characteristic		Total	N0 (%)	N1a (%)	N1b (%)	*P*
Age	Mean ± SD	45.6 ± 14.5	47.4 ± 13.9	43.2 ± 14.4	44.7 ± 15.4	<0.001
	<55	20,780	9,604 (46.2)	7,154 (34.4)	4,022 (19.4)	<0.001
	≥55	7,932	4,369 (55.1)	2,097 (26.4)	1,466 (19.5)	
Race	Black	992	566 (57.1)	256 (25.8)	170 (17.1)	0.001
	White	23,649	11,663 (49.3)	7,548 (31.9)	4,438 (18.8)	
	Other	4,071	1,744 (42.8)	1,447 (35.6)	880 (21.6)	
Sex	Female	21,926	11,683 (53.3)	6,833 (31.1)	3,410 (15.6)	<0.001
	Male	6,786	2,290 (33.8)	2,418 (35.6)	2,078 (30.6)	
T stage	T1	15,661	9,861 (62.9)	4,003 (25.6)	1,797 (11.5)	<0.001
	T2	4,525	1,874 (41.4)	1,823 (40.3)	828 (18.3)	
	T3	7,375	2,053 (27.8)	2,995 (40.6)	2,327 (31.6)	
	T4	1,127	175 (15.5)	423 (37.6)	529 (46.9)	
	TX	24	10 (41.6)	7 (29.2)	7 (29.2)	
M stage	M0	28,393	13,936 (49.1)	9,180 (32.3)	5,277 (18.6)	<0.001
	M1	319	37 (11.6)	71 (22.2)	211 (64.2)	
Multiple distant metastasis	Yes	38	4 (10.5)	7 (18.4)	27 (71.1)	<0.001
	No	211	28 (13.3)	41 (19.4)	142 (67.3)	
	Unknown	70	5 (7.1)	23 (32.9)	42 (60.0)	
Tumor size	Mean ± SD	19.0 ± 15.9	14.5 ± 13.6	22.1 ± 14.8	25.5 ± 19.3	<0.001
	≤1 cm	9,482	6,650 (70.1)	1,773 (18.7)	1,059 (11.2)	<0.001
	>1 cm	19,230	7,323 (38.1)	7,478 (38.9)	4,429 (23.0)	
Grade	I	5,218	2,700 (51.7)	1,522 (29.2)	996 (19.1)	<0.001
	II	986	433 (43.9)	328 (33.3)	225 (22.8)	
	III	187	82 (43.9)	40 (21.4)	65 (34.7)	
	IV	55	15 (27.3)	16 (29.1)	24 (43.6)	
	Unknown	22,266	10,743 (48.2)	7,345 (33.0)	4,178 (18.8)	
Laterality	Bilateral	8,270	3,391 (41.0)	3,002 (36.3)	1,877 (22.7)	0.022
	Unilateral	20,442	9,976 (48.8)	6,582 (32.2)	3,884 (19.0)	
Positive lymph nodes	Mean ± SD	3.0 ± 5.4	0 ± 0	3.7 ± 4.7	9.7 ± 7.6	<0.001
Examined lymph nodes	Mean ± SD	10.7 ± 15.0	4.0 ± 4.8	8.8 ± 9.1	31.2 ± 20.9	<0.001
LNR	Mean ± SD	0.26 ± 0.33	0 ± 0	0.56 ± 0.32	0.40 ± 0.26	<0.001
Radiation therapy	I131 therapy	15,173	4,660 (30.7)	6,411 (42.2)	4,102 (27.1)	<0.001
	No/unknown	12,944	9,167 (70.8)	2,600 (20.1)	1,177 (9.1)	
	beam radiation	595	146 (24.5)	240 (40.4)	209 (35.1)	
Survival months	Median	48	53	44	40	NA
5-year OS (%)	NA	98.3	98.6	97.7	94.6	<0.001
5-year CSS (%)	NA	99.2	99.7	99.2	96.7	<0.001

LNR, lymph node ratio; OS, overall survival; CSS, cancer-specific survival; SD, standard deviation; I, well differentiated; II, moderately differentiated; III, poorly differentiated; IV, undifferentiated (anaplastic); NA, not available.

### The Impact of N Classification on Distant Metastasis

Overall, with the increase of N stage, the prevalence of DM also elevates, including LM and BM. After adjustment for age, race, sex, tumor size, T stage, laterality, radiation therapy, and grade, N1a (OR = 2.23; *P* < 0.001) and N1b disease (OR = 9.22; *P* < 0.001) were significantly associated with DM compared with N0 disease. Similar results were revealed in PTC patients with LM. However, there was no significant difference between N0 and N1a disease in PTC with BM (*P* = 0.69). In contrast, N1b disease was associated with a higher risk of BM compared with N0 disease (OR = 2.78, 95%CI: 1.50–5.36, *P* < 0.001) (**Table 2**). These abovementioned results indicate that LNM significantly increase the incidence of DM, especially for N1b disease.

**Table 2:** The impact of N classification on distant metastasis.

	N stage	Unadjusted			Adjusted^a^		
		OR (95%CI)	*P*		OR (95%CI)	*P*	
DM	N0	Reference			Reference		
	N1a	2.91 (1.97–4.38)	<0.001		2.23 (1.48–3.39)	<0.001	
	N1b	15.06 (10.74–21.72)	<0.001		9.22 (6.42–13.57)	<0.001	
	N1b vs. N1a	5.11 (3.93–6.74)	<0.001		4.14 (3.15–5.50)	<0.001	
LM	N0	Reference			Reference		
	N1a	3.57 (2.06–6.46)	<0.001		2.73 (1.55–5.00)	<0.001	
	N1b	22.91 (14.3–39.3)	<0.001		13.8 (8.41–2.42)	<0.001	
	N1b vs. N1a	6.43 (4.57–9.24)	<0.001		5.07 (3.57–7.35)	<0.001	
BM	N0	Reference			Reference		
	N1a	1.32 (0.64–2.72)	0.446		0.86 (0.41–1.81)	0.690	
	N1b	5.76 (3.25–10.67)	0.001		2.78 (1.50–5.36)	0.001	
	N1b vs. N1a	4.36 (2.40–8.36)	<0.001		3.23 (1.76–6.25)	<0.001	

DM, distant metastasis; LM, lung metastasis; BM, bone metastasis.

a Adjusted for age, race, sex, tumor size, T stage, radiation therapy, grade, and laterality.

### The Impact of N Classification on Distant Metastasis Based on Age Stratification

Patient’s age at diagnosis has been reported to be associated with prognosis (19). The incidence of DM significantly increased with the progress of N disease regardless of older patients or younger patients (*P* < 0.001) (**Supplementary Table S1**). Subsequently, we further explored the influence of N classification on different metastatic sites. In older patients with LM, the results were consistent with the abovementioned observations. In younger patients with LM, N1b disease (OR = 20.45, 95%CI: 8.14–68.77, *P* < 0.001), instead of N1a disease (OR = 2.92, 95%CI: 0.98–10.48, *P* = 0.065), boosts the incidence of LM after adjusting for all confounding variables (**Table 3**). Surprisingly, we discovered that the N classification had no significant effect on BM in younger patients, and only N1b disease (OR = 2.65, 95%CI: 1.32–5.55, *P* = 0.007) elevated the rate of BM in older patients (**Table 3**). These findings imply that more emphasis should be devoted to the impact of LNM on LM rather than BM in younger patients with PTC. However, in older patients, N1b disease significantly increases the risk of both LM and BM.

**Table 3:** The impact of N classification on lung and bone metastasis based on age stratification.

Metastatic sites	Age	N stage	Unadjusted		Adjusted^a^	
			OR (95%CI)	*P*	OR (95%CI)	*P*
LM	<55 years old	N0	Reference		Reference	
		N1a	4.37 (1.55–15.5)	0.010	2.92 (0.98–10.48)	0.065
		N1b	38.19 (15.76–125.78)	<0.001	20.45 (8.14–68.77)	<0.001
		N1b vs. N1a	8.74 (4.97–16.62)		7.02 (3.94–13.46)	<0.001
	≥55 years old	N0	Reference		Reference	
		N1a	4.37 (2.29–8.76)	<0.001	2.88 (1.49–5.87)	0.002
		N1b	20.88 (12.05–39.33)	<0.001	11.83 (6.61–22.86)	<0.001
		N1b vs. N1a	4.78 (3.13–7.53)	<0.001	4.10 (2.65–6.54)	<0.001
BM	<55 years old	N0	Reference		Reference	
		N1a	2.24 (0.55–10.92)	0.269	1.23 (0.29–6.25)	0.779
		N1b	7.18 (2.14–32.36)	0.003	3.46 (0.96–16.56)	0.078
		N1b vs. N1a	3.21 (1.11–10.44)	0.037	2.81 (0.95–9.19)	0.067
	≥55 years old	N0	Reference		Reference	
		N1a	1.44 (0.60–3.35)	0.397	0.76 (0.31–1.79)	0.527
		N1b	6.29 (3.30–12.61)	<0.001	2.65 (1.32–5.55)	0.007
		N1b vs. N1a	4.35 (2.12-9.83)	<0.001	3.50 (1.69–7.98)	0.001

a Adjusted for race, sex, tumor size, T stage, radiation therapy, grade, and laterality.

### Impact of N Classification on Distant Metastasis in Patients With PTMC

Compared with N0 disease, N1a disease does not increase the risk of DM (*P* = 0.069), LM (*P* = 0.058), and BM (*P* = 0.678) after adjusting for age, race, sex, T stage, laterality, grade, and treatment approaches. Meanwhile, the risks of DM (OR = 6.14, 95%CI: 2.25–11.79, *P* < 0.001) and LM (OR = 20.43, 95%CI: 3.37–47.30, *P* = 0.002) were significant higher in patients with N1b disease than patients with N0 disease. Nevertheless, N1b disease did not have a significantly higher risk of BM than those with N0 disease (*P* = 0.550) (**Table 4**). These results reveal that N1a disease does not increase the risk of DM, regardless of LM or BM.

**Table 4:** Impact of N classification on distant metastasis in patients with papillary thyroid microcarcinoma.

	N stage	Unadjusted		Adjusted^a^	
		OR (95%CI)	*P*	OR (95%CI)	*P*
DM	N0	Reference		Reference	
	N1a	4.24 (1.62–11.30)	0.003	2.69 (0.92–8.01)	0.069
	N1b	12.73 (5.59–31.48)	<0.001	6.14 (2.25–11.79)	<0.001
	N1b vs. N1a	3.01 (1.35–7.13)	0.009	2.28 (0.99–5.53)	0.056
LM	N0	Reference		Reference	
	N1a	1.88 (0.087–19.30)	0.608	2.06 (0.90–2.51)	0.580
	N1b	25.30 (6.33–50.78)	<0.001	20.43 (3.37–47.30)	0.002
	N1b vs. N1a	13.49 (2.47–25.04)	0.014	9.89 (1.69–18.75)	0.034
BM	N0	Reference		Reference	
	N1a	0.84 (0.12–4.25)	0.453	0.78 (0.20–3.21)	0.678
	N1b	2.51 (0.40–11.68)	0.271	1.45 (0.32–8.21)	0.550
	N1b vs. N1a	2.12 (0.22–9.31)	0.758	3.25 (0.12–10.34)	0.895

a Adjusted for age, race, T stage, sex, radiation therapy, grade, and laterality.

### Establishment of a Model to Predict Distant Metastasis

**Figure 2:**
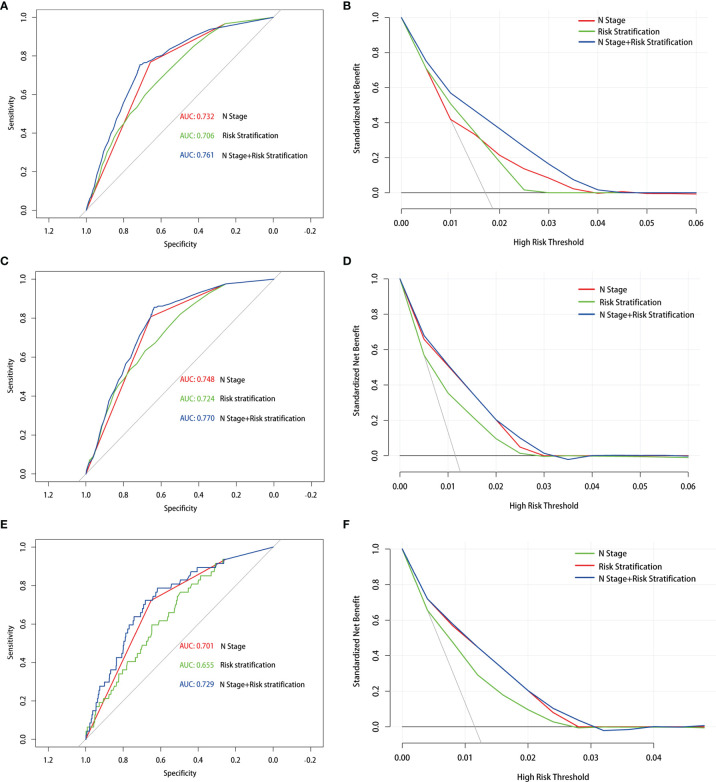
Receiver operating characteristic and decision curve analysis curves with different models in the prediction of distant metastasis **(A, B)**, lung metastasis **(C, D)** and bone metastasis **(E, F)** in papillary thyroid carcinoma patients with 5 or more lymph nodes examined.

In addition, prior studies have reported that the PLNN and LNR played a vital role in predicting DM (20). The clinical implications and appropriate cutoff points have not been comprehensively studied at the same time. To improve the predictive reliability of the PLNN and LNR, the total retrieved lymph nodes less than 5 had been excised. First, we used ROC curves to determine that the cutoff of PLNN and LNR were 3.5 (AUC = 0.725) and 0.286 (AUC = 0.636), respectively (**Supplementary Figure S2**). Thereafter, the enrolled patients were classified into high-risk (PLNN ≥4 and LNR ≥0.286), medium-risk (PLNN >4 and LNR <0.286 or PLNN <4 and LNR ≥0.286), and low- risk (PLNN <4 and LNR <0.286) groups. The statistics model combined N stage with risk stratification and has a well preference in predicting DM (AUC = 0.761). Similar results were shown in PTC patients with LM (AUC = 0.770) and BM (AUC = 0.729). The DCA also revealed that the combined model had an excellent performance in daily clinical practice (**Figure 2**).

## Discussion

This is, to our knowledge, the largest analysis to specifically assess the impact of N classification on different distant metastasis sites. The results have shown that lung was the most frequent metastatic site regardless of N0, N1a, or N1b disease. As the N stage increased, the higher the rate of DM that was identified in the whole cohort even after age stratification. Overall, N1a disease does not increase the risk of DM in PTMCs, and combined N stage with PLNN and LNR has well performance in predicting DM. All these results will help clinicians to develop tailored treatment and follow-up strategies.

Patient’s age at diagnosis has been reported to be a strong prognostic factor in PTC patients (19, 21, 22). Numerous studies have focused on the analysis of prognosis based on age stratification. Our previous research demonstrated that the effect of tumor size on the aggressiveness of PTC varied with the patient’s age (23). Additionally, a study indicated that BRAF mutation was more common in PTC patients over 55 years old (24). It therefore follows that age stratification is critical in assessing unfavorable events (DM, LNM, and gross extrathyroidal extension), although there were some studies about the association between LNM and clinical prognosis in PTC patients (25, 26). To the best of our knowledge, the influence of LNM on DM based upon the age threshold of 55 years has not been fully elucidated. Consequently, this present study filled this gap. Generally, an increase in N stage was associated with a higher risk of DM in both younger and older patients. Unexpectedly, after a more detailed analysis of LM and BM separately, LNM was regarded as a risk factor of LM, but not BM, in younger patients, while LNM was significantly correlated with both LM and BM in older patients. Taken together, the dissimilarities regarding different metastatic sites and variant ages must be considered when exploring the effect of LNM on DM.

More recently, the management of patients with PTMC has become an important issue since over-diagnosis of low-risk PTMCs is a widespread global problem resulting in overtreatment in many cases (27). In this situation, many researchers, including Japanese scholars, suggested that AS rather than immediate surgery could be more beneficial for low-risk PTMC patients (28–31). Nevertheless, the potential risks of AS are increasingly being reported. Approximately 8 to 23.2% of patients experienced an increase in tumor volume during AS (29, 30, 32, 33), and more concerns arise in the occurrence of LNM in PTMC (34–37). Researchers from Kuma Hospital demonstrated that the rate of LNM among PTMC patients who underwent AS could reach 1.2–2.1% (19, 38). In stark contrast to AS, a study conducted in Italy found that none of the low-risk PTMC patients had a lymph node recurrence after surgery (39). Thereafter, Oda et al. observed that the incidence of LNM in AS patients (0.5%) was discrepant to that in immediate surgery patients (0.2%) (40). As demonstrated in these studies, LNM had great implications for treatment decision-making in PTMC. Clinically, LNM seems to be involved in DM (16, 25, 41), an important prognostic factor associated with survival in PTMC (42, 43). Consequently, we sought to ascertain whether the occurrence of central LNM increased the probability of DM during the implementation of AS in PTMC. In this study, it is worth noting that N1a disease did not increase the risk of DM, regardless of LM or BM. Therefore, from the perspective of DM, AS can be chosen for PTMC because it is not too late to perform therapeutic neck lymph node dissection after the occurrence of a central LNM. Accordingly, given the small effect of N1a disease on DM, our study provided clinical evidence to support AS for PTMC patients.

Besides this, the PLNN and LNR were supposed to be strongly associated with the prognosis (44–46). However, the cutoff value was not defined. Schneider DF et al. found that the cutoff values of LNR for predicting CSS and DFS were 0.7 and 0.42, respectively (47, 48). In addition, Machens A et al.suggested that the LNM categories of 0, 1–20, and more than 20 associated better with LM than the current TNM-N categories in PTC (49). Nevertheless, insufficient sample size is a common shortcoming in existing studies. Moreover, the role of PLNN and LNR in predicting the overall DM of PTC was not captured in these studies. Herein 14,400 PTC patients were evaluated, and then it was demonstrated that LNR (cutoff value: 0.286) and PLNN (cutoff value: 4) could be used for risk stratification of DM. Furthermore, the present evidence proved that combined risk stratification with N classification had the best predictive effect on DM, which provides a new perspective for the accurate prediction of DM in PTC.

Although this study revealed numerous important findings, it must be noted that there are several limitations in our study. Firstly, biases, such as selection bias, could have been introduced due to the retrospective design of the study. Secondly, data on the recurrence of DM are lacking in the SEER database, and some other clinical factors associated with DM, such as the size of metastatic nodes, extrathyroidal extension, TERT promoter mutation, multifocality, dose of radioactive iodine therapy, and BRAF V600E, are not available in the SEER database. Thirdly, few patients with PTC presented with DM, limiting the power of the study and making type II errors very likely. Finally, the extension of cervical lymph node dissection and the proportion of prophylactic lymph node dissection in patients are unknown in the SEER database. Nevertheless, data mining from SEER databases can help minimize biases caused by a single-center study. In addition, strict inclusion and exclusion criteria were followed to avoid potential bias in this study.

## Conclusion

In summary, this study based on a large cohort of PTC patients demonstrated that the N stage increased and the higher risks of DM was identified, including LM and BM. However, after age stratification, the incidence of LM, instead of BM, significantly increased with the progress of N disease in younger patients. Meantime, only N1b disease could significantly increase the risk of both LM and BM in older patients. Thus, such discrepancy should be taken into consideration when making treatment strategies. Moreover, N1a disease did not increase the risk of DM, regardless of LM or BM, which indicated that AS was safe for the management of low-risk PTMCs. In addition, we found that combining N stage with PLNN and LNR could individualize the DM estimates for PTC.

## Data Availability Statement

The original contributions presented in the study are included in the article/**Supplementary Material**. Further inquiries can be directed to the corresponding authors.

## Ethics Statement

Ethical review and approval were not required for the study on human participants in accordance with the local legislation and institutional requirements. Written informed consent for participation was not required for this study in accordance with the national legislation and institutional requirements.

## Author Contributions

YD and WW contributed equally to this study. WW and YD conceptualized the project. XL and WJ were responsible for the whole administration. YD and WW analyzed the data and wrote the manuscript. All authors contributed to the article and approved the submitted version.

## Funding

This work was supported by the National Natural Science Foundation of China (grant number 81672885) and the Innovative Foundation for Graduate Students of Hunan Province (grant number 2020zzts259).

## Conflict of Interest

The authors declare that the research was conducted in the absence of any commercial or financial relationships that could be construed as a potential conflict of interest.

## Publisher’s Note

All claims expressed in this article are solely those of the authors and do not necessarily represent those of their affiliated organizations, or those of the publisher, the editors and the reviewers. Any product that may be evaluated in this article, or claim that may be made by its manufacturer, is not guaranteed or endorsed by the publisher.

## Supplementary Material

Supplementary Figure 1 The number **(A)** and percentage **(B)** of different types of distant metastasis in the N0, N1a, and N1b stages of the cohort.

Supplementary Figure 2 Receiver operating characteristic curves with positive lymph nodes number **(A)** and lymph nodes ratio **(B)** in the prediction of distant metastasis.
