# Supplementary figures and images for: Can Cervical Lymph Node Metastasis Increase the Risk of Distant Metastasis in Papillary Thyroid Carcinoma?
Source: Front Endocrinol (Lausanne). 2022 Jun 24;13:917794. doi: 10.3389/fendo.2022.917794 (PMC9263207; doi:10.3389/fendo.2022.917794)

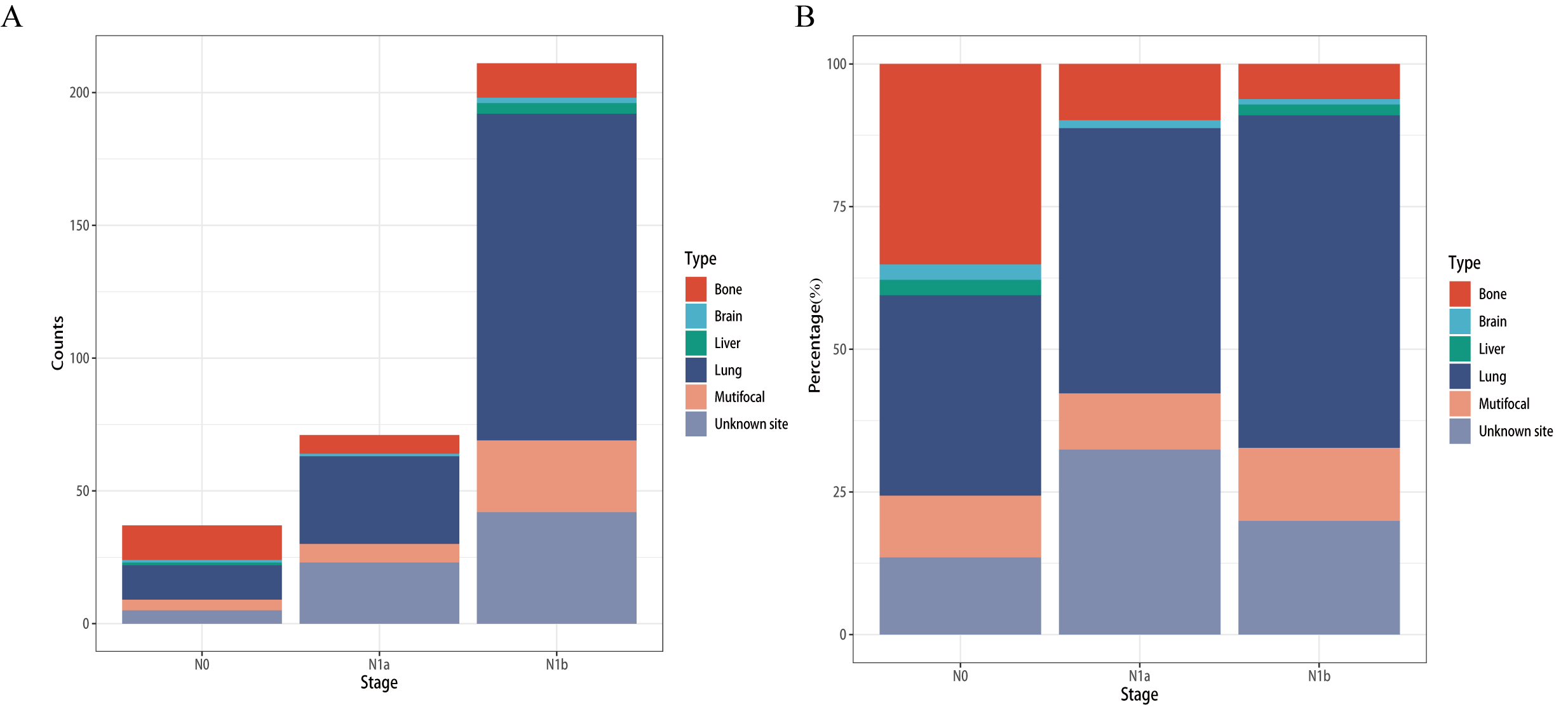

Supplement: Supplementary Figure 1 — The number (A) and percentage (B) of different types of distant metastasis in the N0, N1a, and N1b stages of the cohort. [file Image_1.tif]

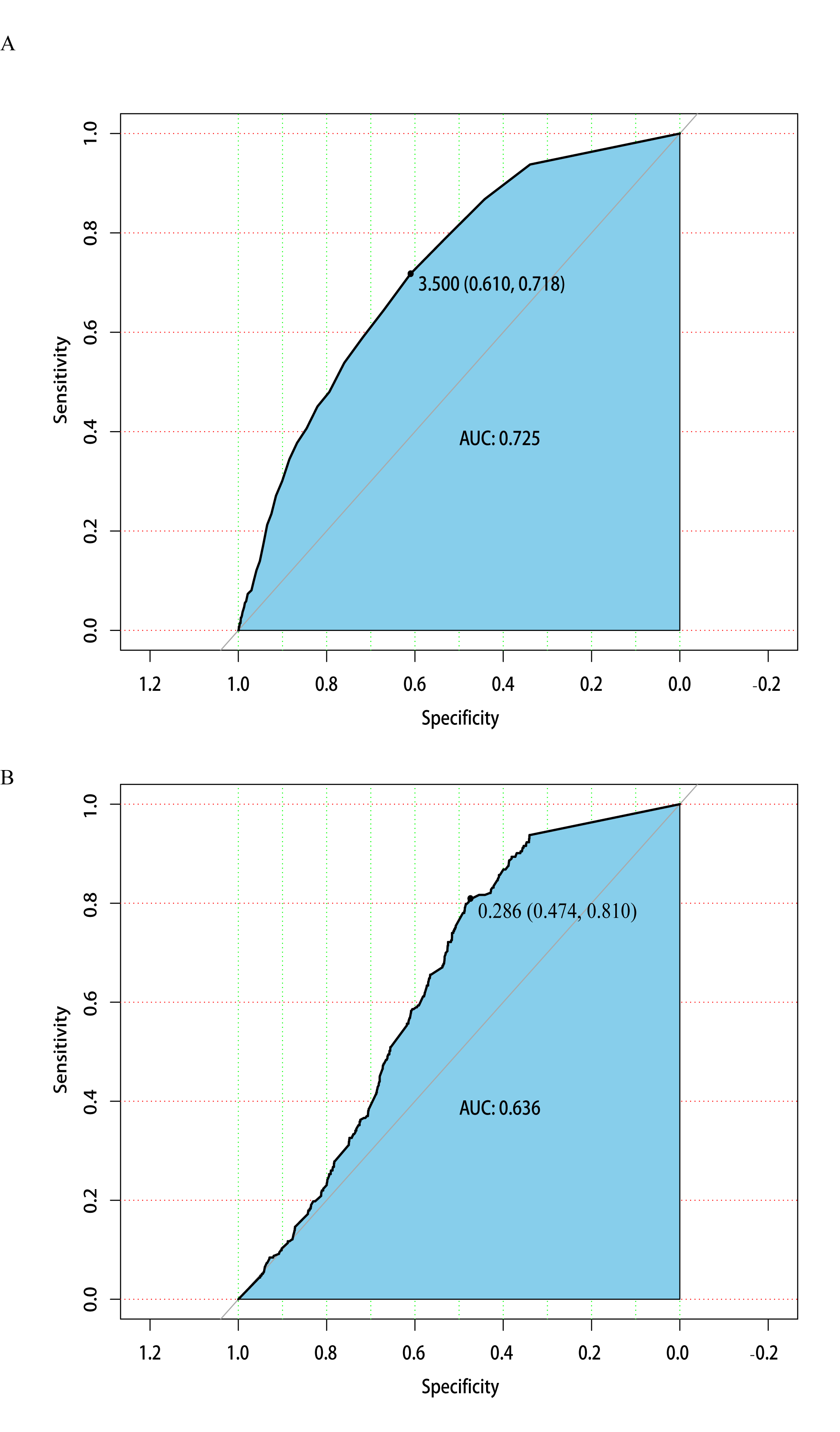

Supplement: Supplementary Figure 2 — Receiver operating characteristic curves with positive lymph nodes number (A) and lymph nodes ratio (B) in the prediction of distant metastasis. [file Image_2.tif]
