# Supplementary material for: Can Cervical Lymph Node Metastasis Increase the Risk of Distant Metastasis in Papillary Thyroid Carcinoma?
Source: Front Endocrinol (Lausanne). 2022 Jun 24;13:917794. doi: 10.3389/fendo.2022.917794 (PMC9263207; doi:10.3389/fendo.2022.917794)
Supplement: Supplementary file 3 [file Table_1.docx]

| Age | N Stage | Unadjusted | | Adjusted^b^ | |
| --- | --- | --- | --- | --- | --- |
|  |  | OR(95%CI) | P | OR(95%CI) | P |
| <55 years old | N0 | Reference | | Reference | |
|  | N1a | 3.93(2.10-7.90) | <0.001 | 2.68(1.40-5.48) | 0.004 |
|  | N1b | 18.92(10.80-36.41) | <0.001 | 10.59(5.83-20.93) | <0.001 |
|  | N1b vs N1a | 4.68(3.21-6.97) | <0.001 | 3.83(2.60-5.74) | <0.001 |
| ≥55 years old | N0 | Reference | | Reference | |
|  | N1a | 3.04(1.83-5.12) | <0.001 | 2.02(1.19-3.48) | 0.009 |
|  | N1b | 15.21(10.02-24.04) | <0.001 | 8.78(5.60-1.43) | <0.001 |
|  | N1b vs N1a | 5.04(3.49-7.46) | <0.001 | 4.39(3.01-6.56) | <0.001 |

Table S1. The impact of N classification on distant metastasis based on age stratification.

^b^Adjusted for race, sex, tumor size, T stage, radiation therapy, grade, bilateral.
