# Supplementary material for: Can Cervical Lymph Node Metastasis Increase the Risk of Distant Metastasis in Papillary Thyroid Carcinoma?
Source: Front Endocrinol (Lausanne). 2022 Jun 24;13:917794. doi: 10.3389/fendo.2022.917794 (PMC9263207; doi:10.3389/fendo.2022.917794)
Supplement: Supplementary file 5 [file Table_3.docx]

#Construction of logistic models

ptc$N<-factor(ptc$N,levels = c("N0","N1a","N1b"),labels=c("N0","N1a","N1b"))

fit1<-glm(group~N+age+Race+Sex+size+T_stage+Radiation+Laterality+grade,data = ptc,family = binomial(link = "logit"))

fit2<-glm(group~N+Race+Sex+size+N+T_stage+Radiation+Laterality+grade,data = ptc,family = binomial(link = "logit"))

fit3<-glm(group~N+age+Race+Sex+N+T_stage+Radiation+Laterality+grade,data = ptc,family = binomial(link = "logit"))

#DCA curves of DM

library(DecisionCurve)

fit1<- decision_curve(DM~N,data = Data, family = binomial(link ='logit'),

thresholds= seq(0,1, by = 0.01),

confidence.intervals =0.95,study.design = 'cohort',

)

fit2<-decision_curve(DM~Risk_stritification,data = Data, family = binomial(link ='logit'),

thresholds= seq(0,1, by = 0.01),

confidence.intervals =0.95,study.design = 'cohort',

)

fit3<-decision_curve(DM~N+Risk_stritification,data = Data, family = binomial(link ='logit'),

thresholds= seq(0,1, by = 0.01),

confidence.intervals =0.95,study.design = 'cohort',

)

plot_decision_curve( list(fit1, fit2,fit3), curve.names = c("N Stage", "Risk","N Stage+Risk"),

col = c("red", "green","blue"),confidence.intervals = FALSE, cost.benefit.axis = FALSE, legend.position = "none")

#DCA curves for lung and bone metastases were similar to those above

#ROC curves of DM

library(ROCR)

fit1<-glm(DM~N,data=ptc,family = binomial(link = "logit"))

fit2<-glm(DM~Risk_stritification,data=ptc,family = binomial(link = "logit"))

fit3<-glm(DM~N+Risk_stritification,data=ptc,family = binomial(link = "logit"))

ptc$roc1<-predict(fit1,newdata = ptc,type = "response")

ptc$roc2<-predict(fit2,newdata = ptc,type = "response")

ptc$roc3<-predict(fit3,newdata = ptc,type = "response")

ROC1=roc(ptc$DM,ptc$roc1)

ROC2=roc(ptc$DM,ptc$roc2)

ROC3=roc(ptc$DM,ptc$roc3)

plot(ROC1,col="red",xlab="1-Specificity")

plot(ROC2,col="green",xlab="1-Specificity")

plot(ROC3,col="blue",xlab="1-Specificity")

#ROC curves for lung and bone metastases were similar to those above
